# Supplementary material for: Maternal Nutrient Excess Induces Stress Signaling and Decreases Mitochondrial Number in Term Fetal Baboon Skeletal Muscle
Source: Biology (Basel). 2025 Jul 17;14(7):868. doi: 10.3390/biology14070868 (PMC12293026; doi:10.3390/biology14070868)
Supplement: Supplementary file 1 [file biology-14-00868-s001.zip › biology-3673214-supplementary---Biology_Statement of Honor 4.pdf]

Statement of Honor

[Biology – Manuscript ID: biology-3673214]

Title: *Maternal nutrient excess induces stress signaling and decreases mitochondrial number in term fetal baboon skeletal muscle*

We, the undersigned authors of the above-mentioned manuscript, hereby declare, in good faith (*bona fides*), that the Western blot results presented in this study are authentic and accurately reflect the original experimental data obtained during the course of this work.

Due to an unforeseen technical failure, the original raw image files from the Western blots were permanently lost, and the unique nature of the biological model used prevents the repetition of the experiments, as no additional samples are available.

Nonetheless, we affirm that all data were generated and analyzed with scientific rigor and integrity. The study was conducted under close supervision, and no data have been manipulated or misrepresented at any stage.

This declaration is made voluntarily and with full awareness of the responsibilities involved in maintaining the highest standards of scientific conduct.

Signed:

Dr. Xu Yan

Dr. Carolina Tocantins

Dr. Mei-Jun Zhu

Dr. Susana P. Pereira

Dr. Min Du
