# Supplementary material for: Maternal Nutrient Excess Induces Stress Signaling and Decreases Mitochondrial Number in Term Fetal Baboon Skeletal Muscle
Source: Biology (Basel). 2025 Jul 17;14(7):868. doi: 10.3390/biology14070868 (PMC12293026; doi:10.3390/biology14070868)
Supplement: Supplementary file 1 [file biology-14-00868-s001.zip › biology-3673214-supplementary.pdf]

Article – Supplementary material

Maternal Nutrient Excess Induces Stress Signaling and Decreases Mitochondrial Number in Term Fetal Baboon Skeletal Muscle

Xu Yan [1,2], Carolina Tocantins [3,4,5], Mei J. Zhu [1,6], Susana P. Pereira [3,4,7,8*] and Min Du [1,9*]

1 Department of Animal Science, University of Wyoming, Laramie, WY 82071, USA;
2 Institute for Health and Sport, Victoria University, Melbourne, VIC 8001, Australia; sean.yan@vu.edu.au (XY)
3 CNC-UC, Center for Neuroscience and Cell Biology, University of Coimbra, Portugal;
4 CIBB, Center for Innovative Biomedicine and Biotechnology, University of Coimbra, Portugal
5 University of Coimbra, Institute for Interdisciplinary Research, PDBEB - Doctoral Programme in Experimental Biology and Biomedicine, Coimbra, Portugal; ctsantos@cnc.uc.pt (CT);
6 School of Food Science, Washington State University, Pullman, WA99164, USA; meijun.zhu@wsu.edu (MJZ);
7 UCIBIO, Applied Molecular Biosciences Unit, Department of Chemistry, Faculty of Science and Technology, NOVA University Lisbon, 2829-516 Caparica, Portugal;
8 Associate Laboratory i4HB-Institute for Health and Bioeconomy, Faculty of Science and Technology, NOVA University Lisbon, 2829-516 Caparica, Portugal;
9 Department of Animal Sciences, Washington State University, Pullman, WA 99164; min.du@wsu.edu (MD)
* Correspondence: Corresponding author: Susana P. Pereira (pereirasusan@gmaill.com) and Min Du (min.du@wsu.edu)

Table S1. List of primary and secondary antibodies used for immunoblotting.

Protein	Name	Supplier	Catalog number
**Primary antibodies**			
TNFα	Tumor necrosis factor alpha	Cell Signaling	3707
NF-κB p65	Nuclear Factor kappa-light-chain-enhancer of activated B cells, subunit p65	Cell Signaling	4764S
Phospho-NF-κB p65 (Ser536)	Phosphorylated NF-κB p65 at the residue Serine 536	Cell Signaling	3033
Phospho-p38 MAPK (Thr180/Tyr182)	Phosphorylated p38 mitogen-activated protein kinase at residues Threonine 180 and Tyrosine 182	Cell Signaling	9211
PGC1α	Peroxisome proliferator-activated receptor γ coactivator 1 α	Cell Signaling	4259S
SIRT1	Sirtuin 1	Cell Signaling	2493S
SIRT3	Sirtuin 3	Cell Signaling	2627S
Cytochrome C	Cytochrome C	Cell Signaling	4272S
β-Tubulin	Beta tubulin	Sigma	T4026
TLR4	Toll-like receptor 4	Santa Cruz Biotechnology	sc-30002
**Secondary antibodies**			
Goat anti-rabbit	-	LI-COR Biosciences	IRDye 800CW
Goat anti-mouse	-	LI-COR Biosciences	IRDye 680

Table S2. List of primers used for transcript amplification.

Mitochondria-related genes	Name	Forward primer	Reverse primer
cox1	Cytochrome C oxidase subunit I	CCCGGAGCCTCTGTGG ACCT	CGGCGGCTAAGACTGG TAGGGA
cox2	Cytochrome C oxidase subunit II	GCAGTGCCTGGACGCC TGAA	TGAAGCTGTGGTTTGCG CCG
PPARGC1A	Peroxisome proliferator-activated receptor-gamma coactivator alpha	ACCCCAAAGGATGCGC TCTCG	CGGTGCGTGCGGTGTCT GTA
PPARGC1B	Peroxisome proliferator-activated receptor-gamma coactivator beta	GCGCTGCTGGACGAAG AGCT	CTCCCCAAAGCAGGTG GCCG
PPARA	Peroxisome proliferator-activated receptor alpha	TGCTGGGTGTTGCTAGC CGC	GACAAGGCTGCAGGCC CCTG
PPARD	Peroxisome proliferator-activated receptor gamma	CAGATGCACCAACGAG GGTCTGG	GGCATCGTCTGGGTCTG AACGC
CREB1	cAMP response element-binding protein	CTCCACAAGTCCAAAC AGTTCAGAT	TCCTTGGCACTCCTGGT GCAT
NOS3	Endothelial nitric oxide synthase	CGGAAGGCTTTTGATCC CCGGG	CATCAGGGCAGCTGCA AAGCTCTC
MFN1	Mitofusin 1	ACCACCCATGCCAAGG AGCGA	AACTTGTTGGCACAGGC GAGC
MFN2	Mitofusin 2	GCGCTCGCTGGTGACGT AGTG	GGCATCATGGACATGG CTGAAGTCC
NRF1	Nuclear respiratory factor 1	GTAGCGCAGCCGCTCT GAGAACTT	GCGAGTCTTCATCAGCA CTCAGCA
SIRT1	Sirtuin 1	AGAGGCAGTTGGAAGA TGGCGGA	GCTCGAGGCCGGGACC ATCTCT
SIRT3	Sirtuin 3	ACATGGCGTTCTGGGGT TGGC	ACCTTTAATAATCGTCC CTGCCGCC
**Inflammation-related genes**			
TNF	Tumor necrosis factor alpha	GCGCCACCACGCCTTCT GT	TGACTGCCGGGCCAGA GGG
TLR4	Toll-like receptor 4	TCCCGGTGTGGCCATTG CTG	TGCCTGCACGACTGCTC AGA
**Reference genes**			
ACTB	Beta actin	CTGGAGGCACCAGCAG GCAC	CAGCGAGGCCAGGATG GAGC
**mtDNA copy number analysis**			
MT-ND1	NADH dehydrogenase subunit 1	CCTATGAATCCGAGCA GCGT	GCTGGAGATTGCGATG GGTA
B2M	Beta-2-Microglobulin	CAGGGCCCAGGACAGT TAAG	GGGATGGGACTCATTC AGGG

Measurement of citrate synthase and β-Hydroxyacyl-CoA dehydrogenase enzyme activity

Muscle samples were homogenized in 10 mM phosphate buffer (0.01 M KH_2PO_4, pH 7.4) in a ratio of 1:20 (w/v) and protein concentration was determined by the Biorad BCA method. For citrate synthase activity, 10 µl of 1:5 diluted muscle homogenate (previously diluted 1:20) were added to 170 µl of phosphate buffer containing Tris buffer (final concentration 0.1 M, pH 8.3), 5,5′-Dithiobis-(2-nitrobenzoic acid) (DNTB) (final concentration 1 mM, in 0.1 M Tris buffer) and oxaloacetate (final concentration 0.01 M, in 0.1 M Tris buffer). Following a background reading, 30 µl of 3 mM acetyl CoA were added to start the reaction. Absorbance was measured on a spectrophotometer (SpectraMax M5, Molecular devices, Sunnyvale, CA) at 412 nm every 30 seconds for 5 minutes at 37°C. To measure β-Hydroxyacyl-CoA dehydrogenase activity, 35 µl of muscle homogenate were added to 190 µl of a solution containing 0.1 M triethanolamine, 5 mM EDTA, 0.45 mM NADH and 15 µl of 2 mM acetoacetyl CoA to initiate the reaction. Absorbance was measured at 340 nm every 30 seconds for 5 minutes at 37°C. Maximum enzyme activity was calculated in micromoles per milligram of protein per minute.
